# Wireless Monitoring of Liver Hemodynamics *In Vivo*

**DOI:** 10.1371/journal.pone.0102396

**Published:** 2014-07-14

**Authors:** Tony J. Akl, Mark A. Wilson, M. Nance Ericson, Ethan Farquhar, Gerard L. Coté

**Affiliations:** 1 Department of Biomedical Engineering, Texas A&M University, College Station, Texas, United States of America; 2 Department of Surgery, University of Pittsburgh, Pittsburgh, Pennsylvania, United States of America; 3 Veterans Affairs Pittsburgh Healthcare System, Pittsburgh, Pennsylvania, United States of America; 4 Oak Ridge National Laboratory, Oak Ridge, Tennessee, United States of America; University of California, Irvine, United States of America

## Abstract

Liver transplants have their highest technical failure rate in the first two weeks following surgery. Currently, there are limited devices for continuous, real-time monitoring of the graft. In this work, a three wavelengths system is presented that combines near-infrared spectroscopy and photoplethysmography with a processing method that can uniquely measure and separate the venous and arterial oxygen contributions. This strategy allows for the quantification of tissue oxygen consumption used to study hepatic metabolic activity and to relate it to tissue stress. The sensor is battery operated and communicates wirelessly with a data acquisition computer which provides the possibility of implantation provided sufficient miniaturization. In two *in vivo* porcine studies, the sensor tracked perfusion changes in hepatic tissue during vascular occlusions with a root mean square error (RMSE) of 0.135 mL/min/g of tissue. We show the possibility of using the pulsatile wave to measure the arterial oxygen saturation similar to pulse oximetry. The signal is also used to extract the venous oxygen saturation from the direct current (DC) levels. Arterial and venous oxygen saturation changes were measured with an RMSE of 2.19% and 1.39% respectively when no vascular occlusions were induced. This error increased to 2.82% and 3.83% when vascular occlusions were induced during hypoxia. These errors are similar to the resolution of a commercial oximetry catheter used as a reference. This work is the first realization of a wireless optical sensor for continuous monitoring of hepatic hemodynamics.

## Introduction

**Figure 1:**
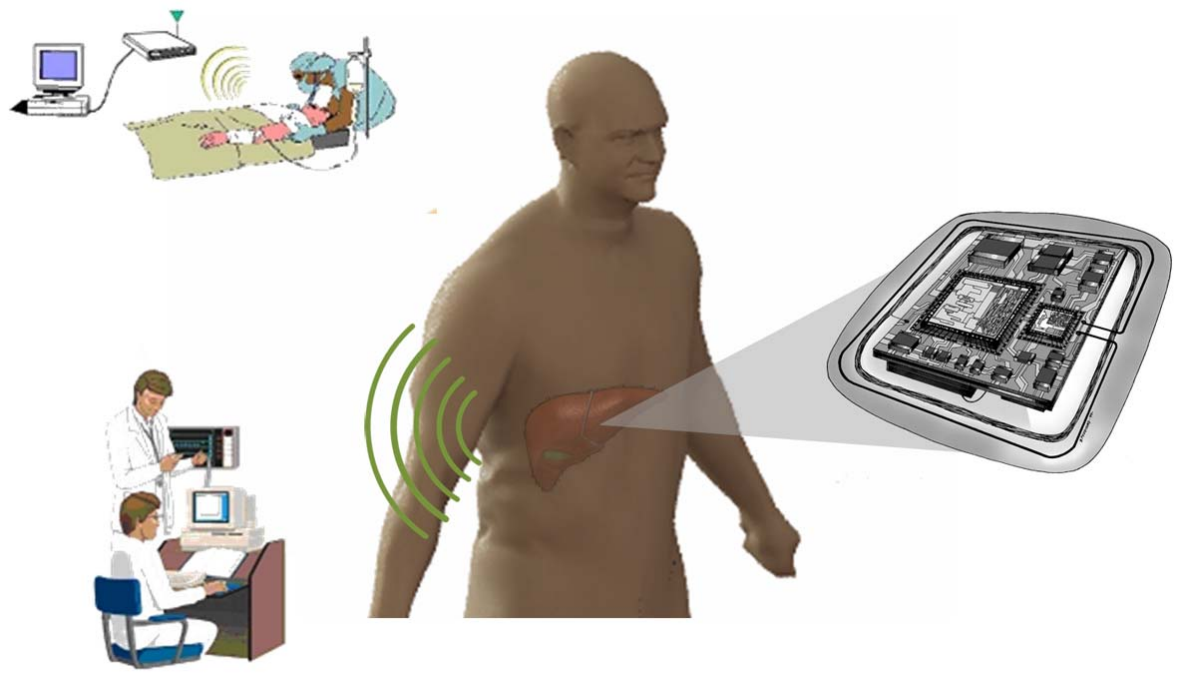
Schematic of the envisioned wireless sensor implanted on the liver.

Since the first successful liver transplant in 1963 [1], this operation has become a standard treatment for end-stage liver disease and acute liver failure. According to the Organ Procurement and Transplantation Network (OPTN) annual report, 28,052 transplants were performed in 2012 and the one year survival rate of the graft was approximately 82% and for the patient 86.5% [2]. The first two weeks post-surgery are associated with the highest technical failure rate [3]. The current standard of care in that period relies on daily blood work and needle biopsies for suspicious results [4]. These tests do not allow timely intervention, and complications are often detected after substantial damage is done to the graft, typically necessitating a second surgery including re-transplantation. Clinical studies by various groups have shown the possibility of using perfusion and/or oxygen consumption as predictors of hepatic graft function post-transplant [5]–[7]. Our group has developed an implantable telemetry system to monitor both tissue perfusion and oxygenation in the parenchyma of the liver which can be used to quantify stress on the tissue related to vascular complications. Detection of such complications at an early stage should allow for timely medical intervention [8]–[11]. With minor adjustments to the hardware, the principle of this sensor can potentially be applied to other tissue models [12]. Figure 1 shows a schematic of the envisioned sensor.

**Figure 2:**
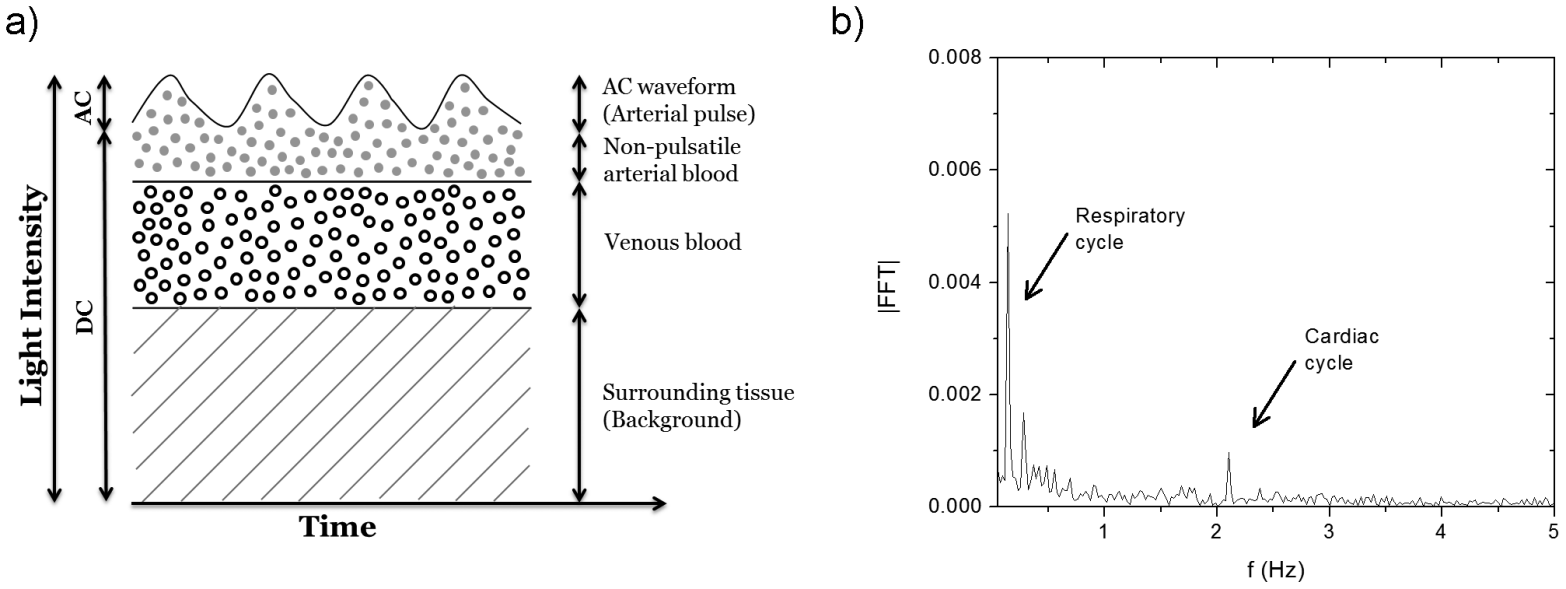
Typical PPG signal. a- Schematic of the collected reflectance signal (Note that the various signal components are not drawn to scale to allow easy visualization). b- Spectrum of the reflectance signal collected with our sensor prior to amplification and with the DC signal omitted.

Our continuous perfusion and oxygen consumption monitoring system is a novel combination of photoplethysmography (PPG) and Near Infrared Spectroscopy (NIRS). It employs Light Emitting Diodes (LEDs) at three wavelengths of light in the near infrared range (735, 805 and 940 nm). The LEDs are time multiplexed, and a single photodetector is used to collect the diffuse reflectance at each of the wavelengths [11]. Because of the pulsatile nature of the arterial blood flow which causes periodic fluctuations in the blood volume contained in the probed tissue, the collected signal has a pulsatile component transduced as an alternating current (AC) by the photodetector and can be used to assess changes in the arterial blood supplying the tissue with oxygen and nutrients. This pulsatile component is typically weak (0.02% to 20%) and needs to be amplified [13], [14]. This waveform constitutes the photoplethysmogram that is used in PPG sensors and pulse oximeters. Figure 2-a shows a schematic of the different components in the collected signal. In addition to the cardiac cycle pulsations, there are other low frequency waveforms that can be detected in the reflectance signal such as the respiratory cycle and some vascular auto-regulation processes [15]. The remainder of the collected signal is a non-pulsatile DC component carrying information about surrounding tissue and the resting blood volume which has a venous and arterial portion. Figure 2-b shows the spectrum of a typical reflectance signal collected *in vivo* with the system described herein prior to the AC amplification. Note that since the DC component is much higher than all the AC components it has been removed in this graph to better visualize the AC peaks as a function of frequency.

Current PPG sensors do not benefit from the information contained in the DC component except for the sole purpose of normalizing the AC signal to account for light coupling, source intensity and other background noise signals. These sensors provide information about the arterial portion of the vascular network. Contrary to PPG sensors, NIRS systems utilize the totality of the reflectance signal to measure concentration changes in the different forms of hemoglobin (oxy-, deoxy-, met- and carboxy-hemoglobin) and other confounder chromophores. However, in most NIRS systems, little attention is given to the pulsatile signal, and the reflectance is used to measure a single value for hemoglobin concentration representing the tissue oxygenation level that is affected by both arterial and venous changes [16]. Some of these NIRS systems employ the pulsatile wave to assess changes in the arterial blood component. To the knowledge of the authors, no NIRS system has successfully separated the venous contribution from the arterial components. As mentioned, separating these two quantities (AC and DC) allows for the quantification of tissue oxygen consumption. This can be used to study the metabolic activity and relate it to tissue stress. Low arterial oxygen levels indicate a deficiency in oxygen supply to the tissue. On the other hand, low levels of venous oxygenation indicate a high consumption rate (low perfusion levels) due to extraction of oxygen by the tissue. These two levels coupled with a relative perfusion measurement can be used to detect hemodynamic complications and identify potential causes. The sensor presented in this paper aims to simultaneously measure venous oxygen saturation, arterial oxygen saturation, and perfusion levels to provide physicians with a more complete picture of graft hemodynamics in order to assess graft function intra- and post-operatively.

The current standards for monitoring tissue perfusion levels are laser Doppler flowmetry (LDF), transit-time ultrasound and thermal diffusion [17]–[19]. Although these techniques have been shown to be reliable in measuring perfusion changes, they lack any sensitivity to blood oxygen levels. Thus, optical spectroscopy offers a significant advantage over these techniques because of the dependence of the hemoglobin extinction spectrum on its oxygenation state [20]. Multiple groups have shown the efficacy of NIRS in monitoring hepatic hemodynamics [21], [22]. In this paper, we report the first *in vivo* testing of a wireless optical sensing system to monitor venous and arterial oxygenation coupled with perfusion changes in liver tissue. This device is a first step toward a fully implantable sensor for graft monitoring.

## Materials and Methods

### A. Instrumentation

The system developed for these studies consists of three sensors. Two of the sensors are equipped with vascular probes to monitor the vessels supplying blood to the liver, the hepatic artery (HA) and the portal vein (PV). The third probe was used to monitor changes in the hepatic parenchymal tissue. The HA and PV probes served as reference measurements and are not required for the final application since the measurements and calculations can be performed with the parenchymal probe alone. In addition, vascular probes are not favored by surgeons due to the complications they can cause by disturbing blood flow or injuring blood vessels during removal. The system consists of four printed circuit boards, three of which are identical sensor interface boards each having the full functionality of a sensor including three programmable amplitude, time-multiplexed LED drives, and synchronized detector signal amplification, dark current subtraction, filtering, and digitization. The fourth board includes the microcontroller unit that communicates with and controls the three sensors via the sensor interface boards. The microcontroller also communicates wirelessly with a relay unit connected to a laptop computer. The computer sends the acquisition parameters, provides the visual interface, and allows further data analysis and storage. The computer graphical user interface was developed in Python to control the sensors, and allow the user to visualize the data in real time. All electronics and a rechargeable lithium-ion battery (BatterySpace.com, part# CU-MM184) were encapsulated in a custom plastic box (90.17×80.00×62.23 mm^3^) built using a 3D printer and coated with polydimethylsiloxane (PDMS). The three probes were constructed using multi-wavelength LEDs (Epitex L660/735/805/940-40B42) and a silicone photodetector (Hamamatsu S2833-01) soldered to a custom printed circuit board. Note that these LEDs contain four wavelengths in a single package; however only three wavelengths (735, 805, and 940 nm) were used in our sensors. The probes were also coated with PDMS to avoid any leakage to the electronics. The source to detector separation was set to 4 mm edge to edge (∼8.7 mm center to center) [8]. Although perfusion and oxygenation information can be obtained using only two wavelengths, we added a third wavelength to be able to account for artifacts on the signal such as motion and other confounding chromophores. This feature was not used in the processing of the data presented herein since these studies were performed in a controlled environment. More details about the probes' geometry and the system design can be found in the supporting information (Figure S1). Figure S2 in the supporting information shows a picture of the system..

### B. Data processing

Each of the sensors extracts an AC signal from the collected diffuse reflectance (band-pass f_3dB_ ∼0.7 and 24.7 Hz) and amplifies it by a gain specified by the user (1 to 96 X). Both the DC and AC signals are transmitted to the data acquisition relay unit and saved on a computer for further processing.

The signal processing used to extract oxygenation levels can be divided in three parts. First, the normalized pulsatile signal (AC/DC) on two of the wavelengths was used to measure the oxygenation of the pulsatile blood flow similar to pulse oximetry. This measurement tracks the oxygenation of the hepatic blood supply. Second, the DC signal was processed similar to NIRS signals which track tissue oxygen saturation. Both venous and arterial blood contribute to tissue oxygenation. Last, the venous oxygen saturation was extracted from the tissue oxygenation levels using the already calculated arterial oxygenation. These three steps are explained in more details in the following three sections.

#### AC processing

Fourier processing was used to measure the amplitude of the pulsatile wave. The Fast Fourier Transform (FFT) was calculated for 25 s data intervals using software developed in MATLAB (Mathworks, Inc.). The software detects the frequency peak that corresponds to the cardiac cycle and uses it as the amplitude of the AC signal. The frequency of that peak was used to measure the heart rate in beats per minute (60*f_peak_).

Using the AC amplitude at the 735 and 940 nm wavelengths, we calculated the modulation ratio R that is typically used in pulse oximeters to assess oxygen saturation. This quantity requires a calibration curve to produce quantitative oxygen saturation results. To calibrate our sensor we fit the data to a calibration model in the form shown in equation 1-b. Although the fitting parameters (a, b, c, and d) can be estimated theoretically, previous work has shown that the practical calibration curve is different from the theory that is simplified and does not account for the effects of scattering [23]. However, the general form of the equation still holds.
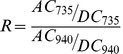
(1a)

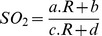
(1b)The quantity (R) is a measure of the ratio of absorbance at the red and NIR wavelengths of the pulsatile perfusion. Due to the compliance of the tissue, blood flow loses its pulsation on the venous side. Thus R is used to follow the changes in the oxygen supply (i.e. arteries and the arterial side of the capillary network).

#### DC processing

The DC signals were processed using the typical equations employed for NIRS signals [24]. The pathlength factor was estimated using the theoretical equation reported by Boas *et al.* [24]. These equations are used to derive the change in concentration of oxyhemoglobin, deoxyhemoglobin, and total hemoglobin (ΔHbO_2_, ΔHb, and ΔHbT respectively). The change in total hemoglobin concentration (ΔHbT) tracks perfusion. To obtain a measure of the change in oxygenation, we used the hemoglobin oxygenation index (ΔHbD = ΔHbO_2_−ΔHb) [25]. During perfusion changes, ΔHbO_2_ and ΔHb vary similarly and the difference (ΔHbD) remains unaltered. However, for oxygenation changes, ΔHbO_2_ and ΔHb vary in opposite directions leading to a change in the hemoglobin oxygenation index (ΔHbD).

#### Venous oxygenation

**Figure 3:**
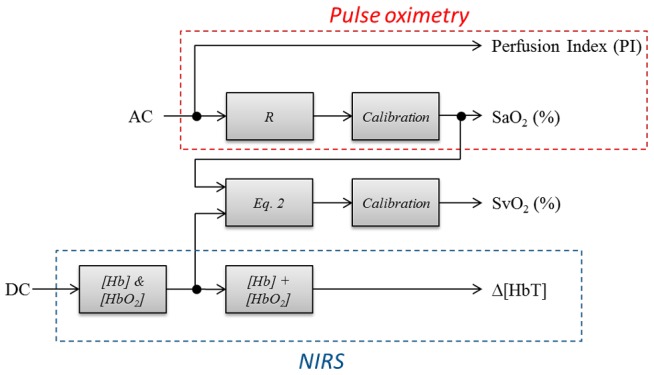
Flow chart of the signal processing. This was employed to compute the oxygen saturation at the arterial and venous side along with perfusion changes from the measured AC and DC signals.

The values calculated using the DC levels as described above represent tissue oxygenation which may be affected by either changes in oxygen supply (arterial side) or in oxygen consumption (venous side). It is of paramount importance to separate the two contributions to be able to possibly diagnose the cause of complications when they occur. Most previous reports employing NIRS measured, or assumed a constant percent contribution of, venous and arterial components to the collected signal [16], [26]. This ratio is different for various types of tissue and probe geometries. If this ratio is determined it can be used to extract the venous signal contribution to the DC levels. To accomplish that we used Multiple Linear Regression (MLR), and the measured venous oxygenation was fit to the linear combination of the DC measured changes (ΔHbD) and the oxygen saturation of the supply (equation 2). Figure 3 shows a flow chart of the signal processing.
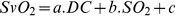
(2)


### C. Animal study protocol

The animal study was performed on two swine (a 21 kg male and a 27 kg female). Prior to anesthesia, the animals were premedicated with Telazol (5–10 mg/kg intramuscularly, im) and the analgesic Buprenorphine (0.01–0.05 mg/kg, im). To induce anesthesia, the animals were administered 3–4% Isoflurane in oxygen at 3 L/min via a face mask. An endotracheal tube was inserted into the trachea, secured in place and connected to a mechanical ventilator (8–12 BPM and tidal volume of 5–10 mL/lb). Isoflurane (0.5–4%) was administered in oxygen to maintain anesthesia. A laparotomy was performed to expose the liver and its vasculature. Transit-time ultrasound probes were placed on the hepatic artery (Transonic Systems, cat# MA4PSB) and the portal vein (Transonic Systems, cat# MA10PSB) to monitor flow changes. An oxygenation catheter was placed in the aorta via the iliac artery (Edwards Lifesciences, cat# XA3820HKCDC) and connected to a Vigilance Monitor (Edwards Lifesciences). Another oxygenation catheter was placed in the inferior vena cava at the level of the hepatic veins to monitor venous oxygenation changes. Two laser Doppler flowmeter probes were placed on the parenchyma to monitor tissue perfusion changes. A catheter was placed in the femoral artery to monitor arterial pressure. Throughout the experiments, vital signs (body temperature, SpO_2_, heart rate, blood pressure, etc.) were monitored closely and recorded. All reference equipment was connected to a custom built data acquisition system and saved for further processing. Similarly, three probes from the optical telemetry system were placed on the HA, PV, and the liver parenchyma. All probes were secured using 5/0 polypropylene sutures. Note that the HA and PV probes were placed for reference; however, no vascular probes will be required for the final application. Vascular occluders (Harvard Apparatus, part.# PY2 62-0111, -0113, and -0117) were placed on the HA and PV to be able to alter hepatic flow and perfusion. Hypoxia was induced by inhalation of low oxygen content mixtures. The wireless sensor electronic unit was placed outside the body next to the animal on the surgical table and tethered to the probes. The relay unit was connected to a laptop placed approximately 4 feet from the surgical table. Data were collected intermittently prior, during, and after any flow or oxygenation perturbation. At the end of the experiment, while still anesthetized, the animal was euthanized with a barbiturate derivative solution administered intravenously (80–120 mg/kg). Finally, livers were extracted and weighed (465 and 525 g for the 21 and 27 kg animals respectively) to convert the flow measurements (mL/min) into an average tissue perfusion measure (mL/min/g of tissue). All studies were performed under an animal use protocol (AUP #2010-257) approved by the Institutional Animal Care and Use Committee at Texas A&M University.

### D. Reference Measurements

The liver has a complex vasculature that is supplied by two different vessels: the hepatic artery (HA) and the portal vein (PV). The HA supplies approximately 25% of nutrients and total blood flow but it is rich in oxygen and delivers approximately 75% of the total liver oxygen supply. The PV is part of the venous system but supplies blood to the liver. The portal venous blood is rich in nutrients but relatively poor in oxygen, and it supplies roughly 75% of liver nutrients along with 25% of its oxygen. Thus, the supply oxygenation in the liver tissue is not that of the artery alone, but it is the addition of the contribution of the PV and the HA. In the remainder of this manuscript we will refer to this quantity as the mixed oxygen saturation (MOS) as defined in equation 3. Due to the limited exposed space on the PV (few centimeters) and the required flow probes and vascular occluders placed on that vessel, we could not insert a catheter into the PV to monitor its oxygenation. We assumed it to be equal to the non-hepatic venous oxygenation for the purpose of our calculations.
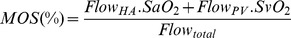
(3)Note that MOS represents the oxygen supply to the liver and will be compared to the oxygen saturation predicted by the pulsatile AC signal as discussed earlier (Figure 3).

Similarly, total hepatic flow is the sum of PV flow and HA flow. In addition to the Laser Doppler Perfusion monitor, we used the total flow as measured by two Transit-Time vascular flow monitors to track tissue perfusion as described by equation (4) below:
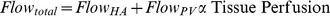
(4)Two different data collection procedures were used on the two animals. For the first study, we induced hypoxia without imposing any change in the hepatic flow to test the ability of the sensor to track oxygenation changes. The second study began with four consecutive hepatic artery occlusions, followed by three portal vein occlusions. These occlusions were performed at normal systemic oxygenation levels. Hypoxia was induced afterwards, and vascular occlusions (HA and PV) were performed again at low systemic oxygenation levels. All occlusions were brief (less than 1 minute for full occlusion) and were performed in gradual steps. Although the inhaled oxygen level was not changed during occlusions, the hepatic oxygen saturation is expected to be altered due to an increased hepatic oxygen extraction ratio and a change in the relative flow (see the MOS equation above) of the HA (high oxygen content) and PV (low oxygen content).

## Results and Discussion

**Figure 4:**
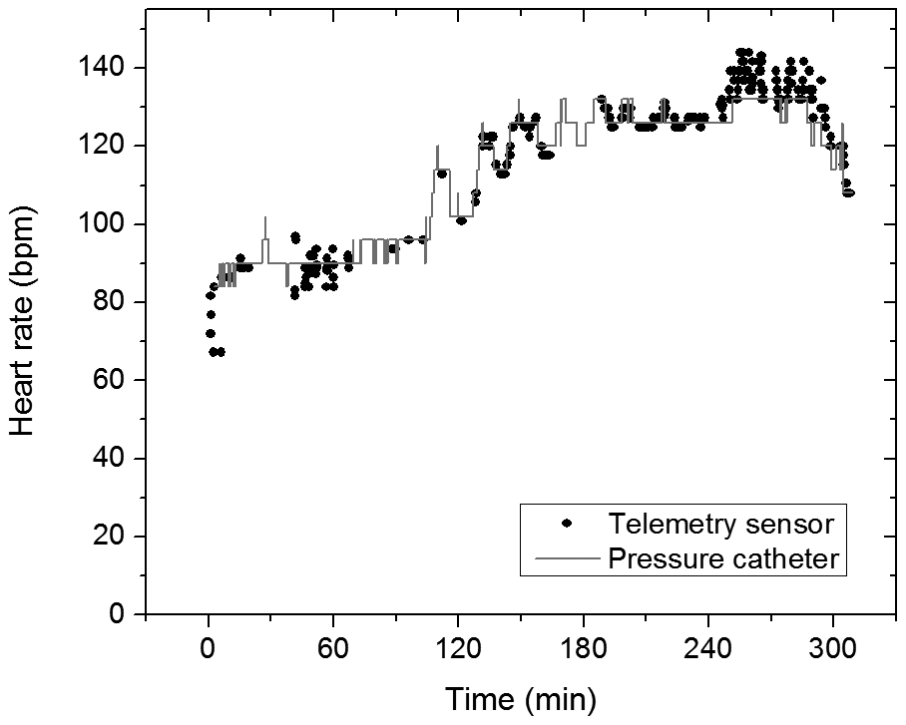
Heart rate measurements. Data from the arterial pressure catheter (grey) and the telemetry sensor (black).

To verify that the pulsatile signal is tracking the cardiac cycle, we looked at the cardiac cycle peak detected by our system and compared it to the heart rate measured by the arterial pressure catheter. This was performed on data from both animals and the detected heart rate was accurate with a Root Mean Square Error (RMSE) of 3.9 bpm (0.065 Hz). Some of this error is due to the difference in the integration time between the telemetry sensor (25 s) and the pressure catheter (2 s). Figure 4 shows the heart rate throughout the study as measured from the arterial pressure catheter and the FFT of the PPG signal measured with the telemetry sensor.

### A. Oxygenation data

**Figure 5:**
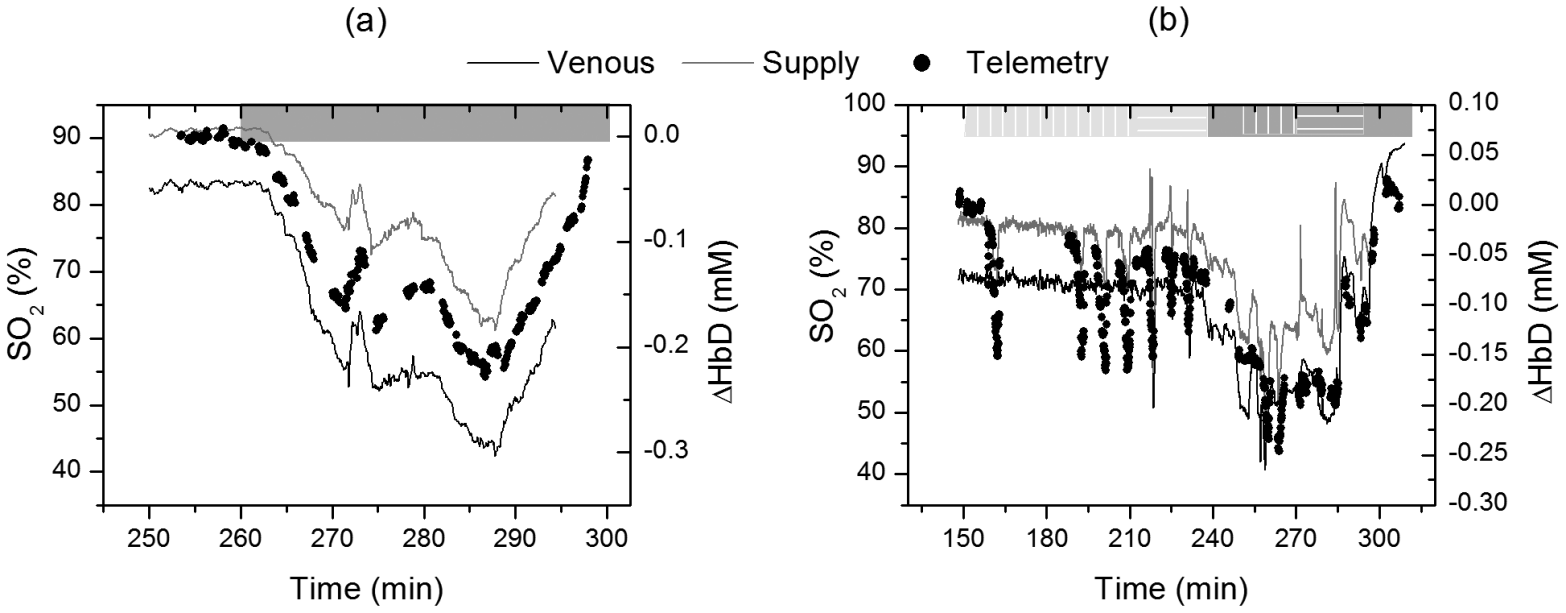
Oxygenation changes as measured by the sensor and the oximetry catheters. Hemoglobin oxygenation index (right axis) measured by the optical telemetry system versus venous and mixed oxygen saturation (left axis) for study 1 (left panel) and 2 (right panel). Venous oxygenation is measured by the oximetry catheter placed in the vena cava while the supply oxygenation is the weighted average of the HA and PV oxygenation described by equation 3.

To track oxygenation changes, the hemoglobin oxygenation index (ΔHbD) was computed, as obtained from the measured DC levels, which, as discussed in the Materials and Methods section, is a measure of tissue oxygenation affected by both arterial and venous oxygen saturation levels. This level is compared to both oxygen supply (MOS) and venous oxygenation (SvO_2_) from the two different studies as shown in Figure 5. The raw data from the oximetry catheters are shown in the supporting information (Figure S5). As described earlier, for the first study (Figure 5-a) we induced hypoxia and we did not alter perfusion. However, in the second study (Figure 5-b) we had multiple occlusions of the HA and PV at various levels of oxygen saturation during hypoxia. Vascular occlusions have been shown to alter tissue perfusion and oxygenation in liver tissue [21]. In the following figures, hypoxia periods are indicated by a dark grey box on the upper horizontal axis. Note that the recovery period from hypoxia is also included in the grey boxed region. Vertical white lines indicate segments where one or more hepatic artery occlusions were performed while horizontal white lines indicate where one or more portal vein occlusions were performed. The occlusion periods include multiple occlusions and baseline readings in between.

**Figure 6:**
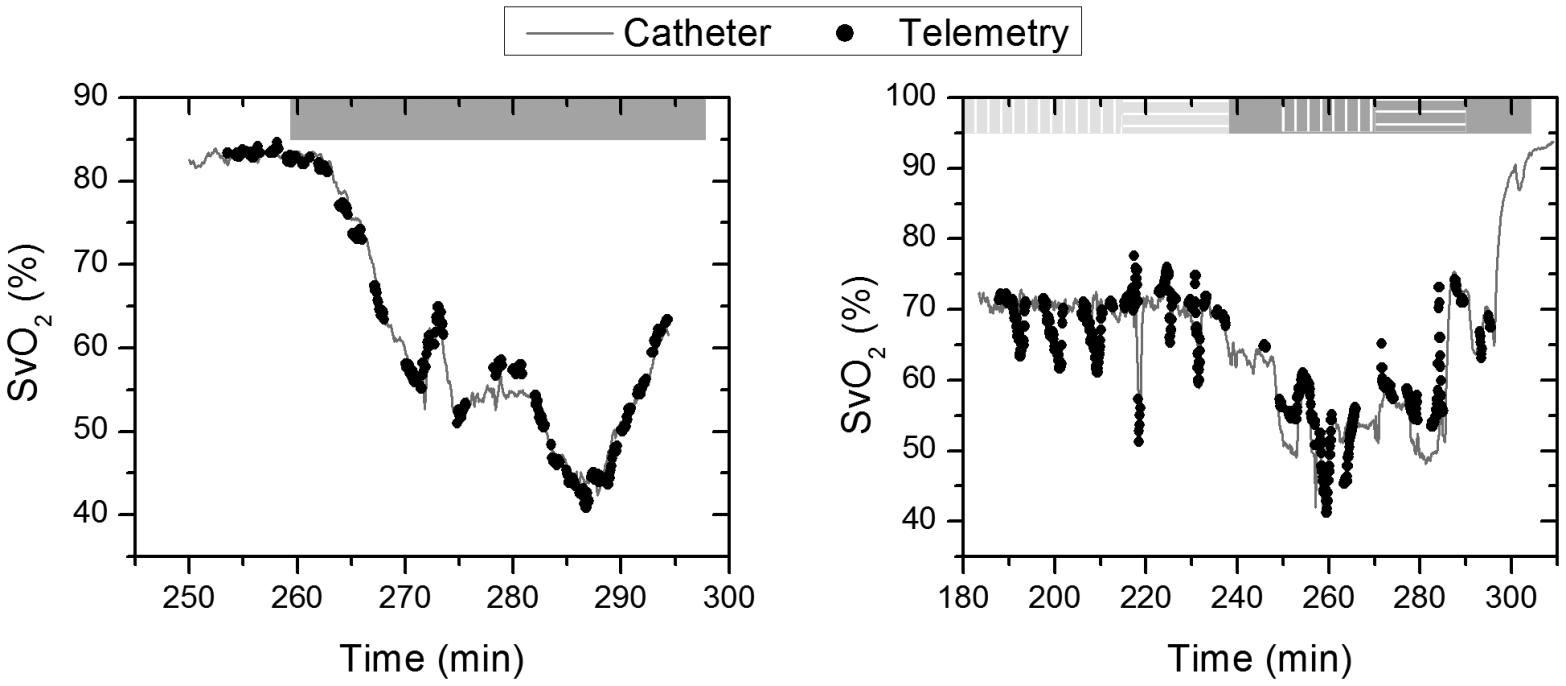
Venous oxygen saturation. Data from the telemetry sensor (black dots) and the central venous catheter (grey line) for study 1 (left) and 2 (right).

Figure 5 indicates that the measured hemoglobin oxygenation index is tracking oxygenation changes. This measure was obtained from the collected DC signal that is probing both the HA/PV supply and the post-hepatic venous components. To verify that this measure contains oxygenation information about both supply and hepatic venous blood we correlated the measurements to the reference data obtained from the oxygenation catheters and flow meters. Although ΔHbD correlated well with both supply and venous oxygenation (data shown in supporting information, Figure S6), it correlated best with a combination of the two using multiple linear regression (MLR) analysis described by equation 5. The coefficient of determination (R^2^) was 0.99 for study 1 (no vascular occlusions) and 0.80 for study 2 (vascular occlusions at multiple levels of oxygenation) respectively. This corresponds to a root mean square error (RMSE) of 1.39% and 3.93% respectively.
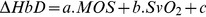
(5)Having the calibration coefficients (a, b, and c) from the MLR, the mixed oxygen saturation (MOS) measured by the gold standards, and the hemoglobin oxygenation index (ΔHbD) measured by the telemetry system, equation 5 was used to compute venous oxygen saturation (SvO_2_). Figure 6 shows the predicted venous oxygenation and the measured oxygenation by the venous catheter as a function of time. Note that in study 2, occlusions are performed on the hepatic artery and are always seen as a decrease in the measured venous oxygenation by our probe. However, this decrease is not detected by the venous catheter during the first three occlusions because the catheter was measuring hemoglobin oxygen saturation in the vena cava rather than the hepatic veins. Thus, the first three occlusions performed on the hepatic artery caused a decrease in the hepatic venous oxygen saturation as shown by our probe but did not have a substantial effect on the systemic venous oxygenation, where the venous catheter is measuring, since the HA flow is relatively low (7% of total cardiac output) compared to the portal vein flow (22% of total cardiac output).

**Figure 7:**
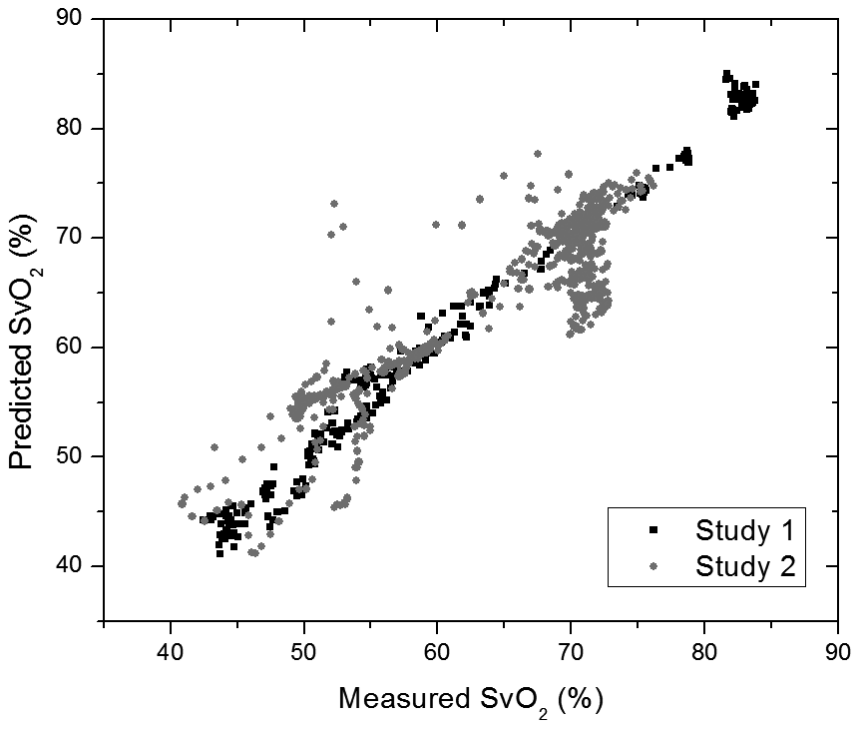
Scatter plot of the predicted (telemetry) versus measured (catheter) venous oxygen saturation for both studies (1: black & 2: grey).

The data from both experiments are shown on the scatter plot of figure 7. Note that the data from study 2 have a higher RMSE (3.93%) compared to study 1 (1.39%). This increase is mainly due to the occlusions performed in that study, and part of this perceived error is from the reference measurements, and not our system, because it was probing central venous oxygenation and not the hepatic vein.

**Figure 8:**
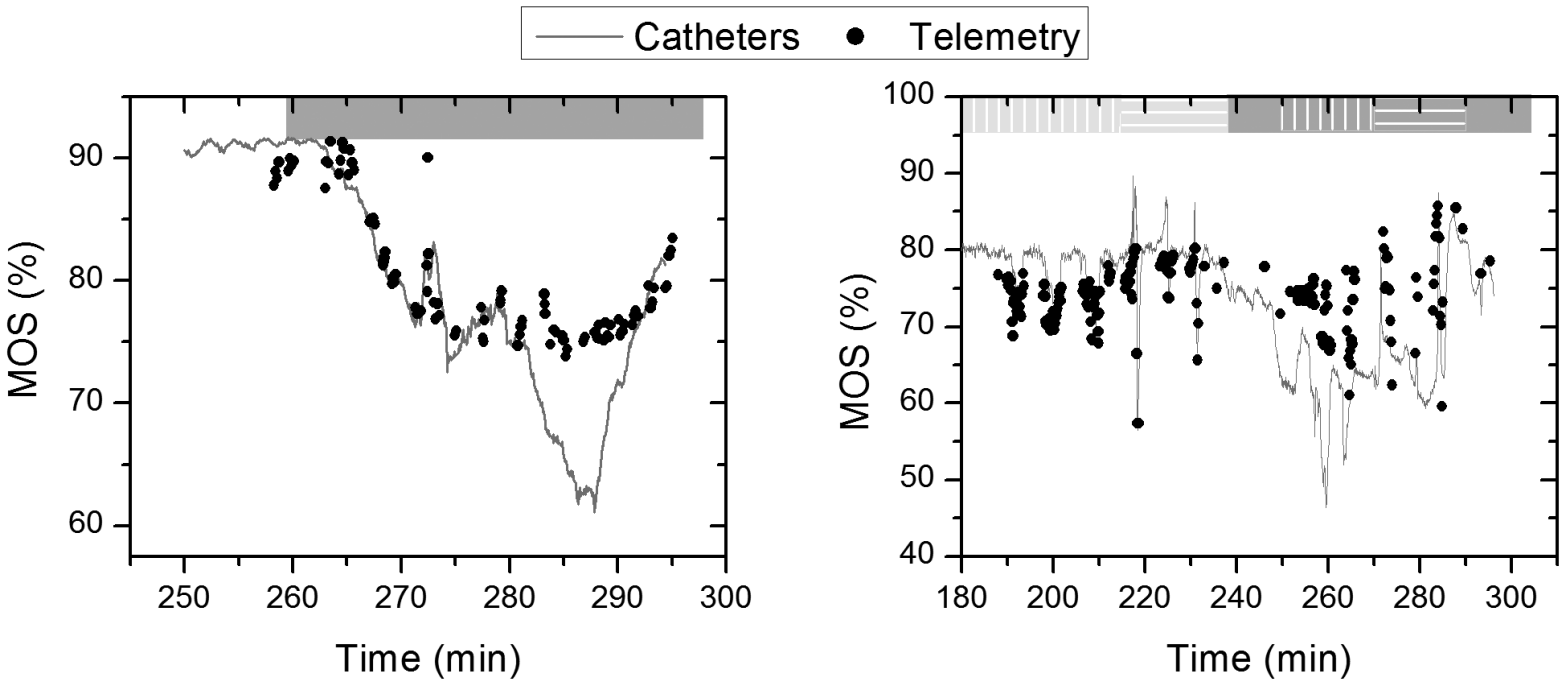
Mixed oxygen supply (MOS) measured by the telemetry sensor (black dots) and the reference equipment (grey line) for both studies (1: left, & 2: right).

The data shown in Figures 4 and 5 use the supply oxygen saturation levels obtained from the catheters to extract the venous oxygenation from the DC levels. However, as described earlier, we wanted to determine the supply oxygenation levels from the pulsatile signal and avoid using any additional reference measurements. To do so, the modulation ratio (R) was calculated, and measurements were calibrated as discussed in the materials and methods section. The data from each experiment were calibrated separately. These measurements were compared to the Mixed Oxygen Saturation (MOS) described earlier. Figure 8 shows the measured MOS and the reference data from the catheters and the flowmeters computed using equation 3. The modulation ratio was able to predict the MOS except when the oxygen saturation dropped below 75%. This is a known problem of pulse oximeters and is mainly due to the high attenuation of the red wavelength (735 nm) for low oxygenation levels. If needed, this issue can be resolved by optimizing the sensor design (illumination wavelength, amplification, etc.) to operate for low oxygen saturation levels [27]. During study 2, the oxygen saturation during vascular occlusions dropped to as low as 56%, and the sensor was still able to measure it accurately. We believe this is due to the decreased absorbance as a result of the perfusion decrease that allowed the red wavelength (735 nm) to still be measured accurately.

**Figure 9:**
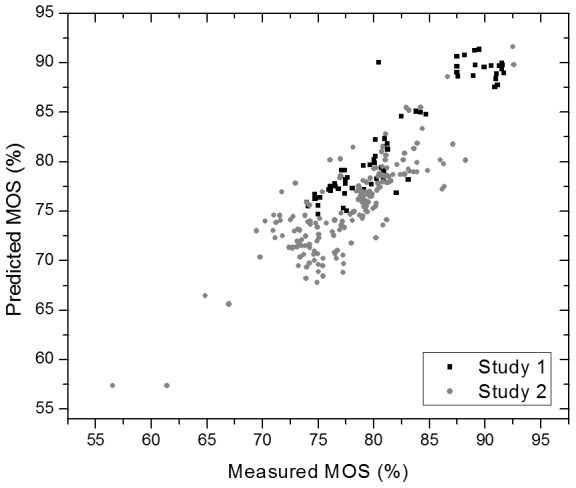
Scatter plot of the predicted vs. measured MOS for study 1 (black) and 2 (grey).

For oxygenation levels above 72% (for normal perfusion levels) and 55% (for low perfusion levels) the calculated modulation ratio from the pulsatile signal was able to predict oxygenation changes with an RMSE of 2.19% and 2.82% for study 1 and 2 respectively. Figure 9 shows the corresponding scatter plot.

**Figure 10:**
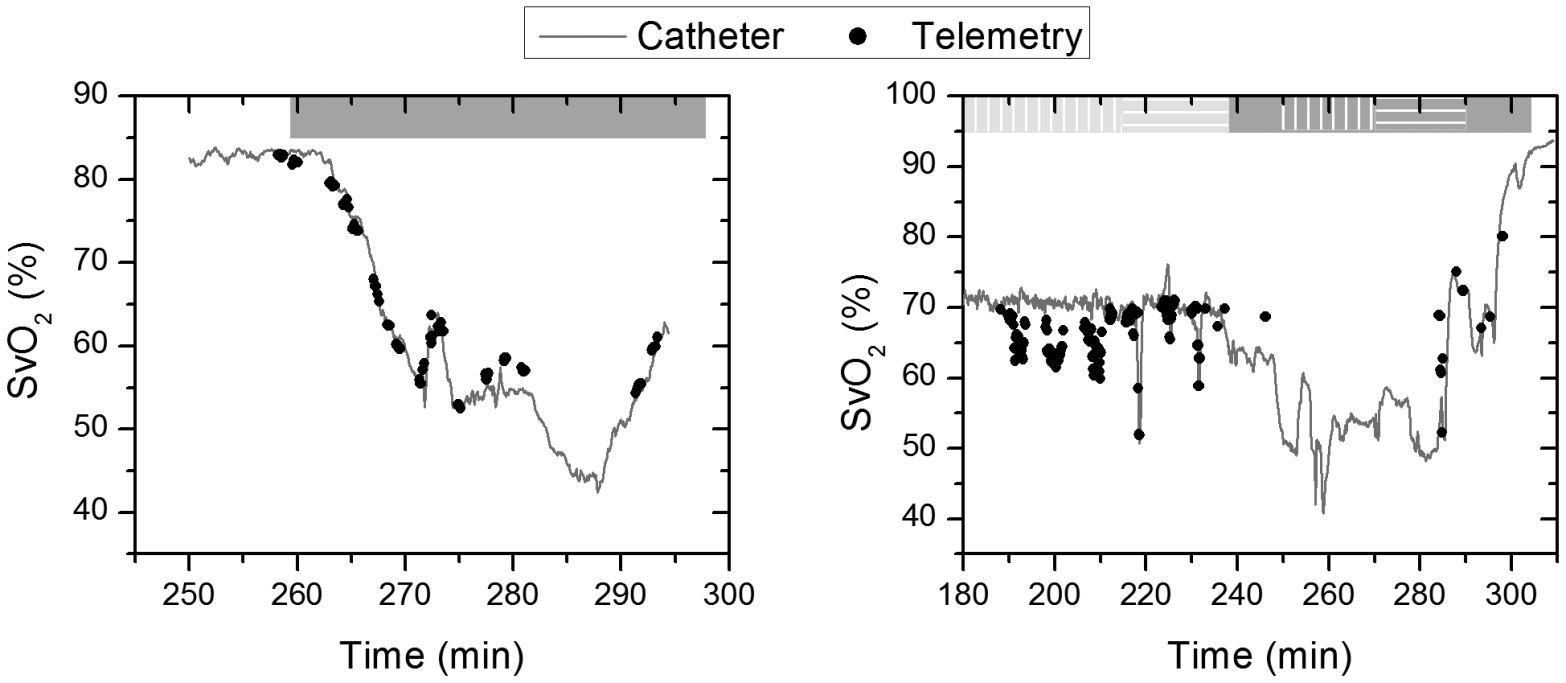
Predicted venous oxygen saturation by combining the DC NIRS measurements with the AC pulse oximetry measurements. Note that the missing values correspond to the periods where MOS dropped below 72% and, because of the previously reported problem with signal from the red wavelength, could not get reliable pulse oximetry measurements. Study 1 & 2 are shown in left & right panel respectively.

These predicted MOS levels can be used with equation 5 to predict venous oxygenation SvO_2_ without the need for a reference measurement. Because the pulsatile signal could not predict data for very low oxygen saturations, we tested this concept on all other parts of the data, and we obtained the measurements shown in Figure 10. For study 1, we were able to measure venous oxygen saturation with an RMSE of 1.17% (R^2^ = 0.986) while for study 2 the RMSE was higher at 3.44% (R^2^ = 0.1). This increased prediction error is mainly due to the changes measured during HA occlusions that are not reflected in the central venous oximetry catheter measurements. However, we believe that these changes likely reflect variations in the hepatic venous oxygenation.

### B. Perfusion data

One of the important features of the presented sensor is the ability to track perfusion and oxygenation changes simultaneously. To track perfusion changes, we measured the change in tissue total hemoglobin concentration (ΔHbT) as described in the Materials and Methods sections. After analyzing the laser Doppler flowmetry (LDF) data from the commercial system, we found that the LDF signal correlated with changes in HA flow but not PV nor total flow (R-square = 0.8, 0.1, and 0.5 respectively) measured by vascular transit-time ultrasonic flowmeters. These data are shown in detail in Figures S3 & S4. We believe that the LDF was probing a branch of the HA and was not tracking tissue perfusion. Because the LDF measurements reflected the HA flow and not tissue perfusion, they were not used as a reference for parenchymal perfusion. Instead, the total hepatic flow measured by the addition of the signal from two transit-time ultrasound flowmeters (HA & PV) was used as the reference for parenchymal perfusion measurements.

**Figure 11:**
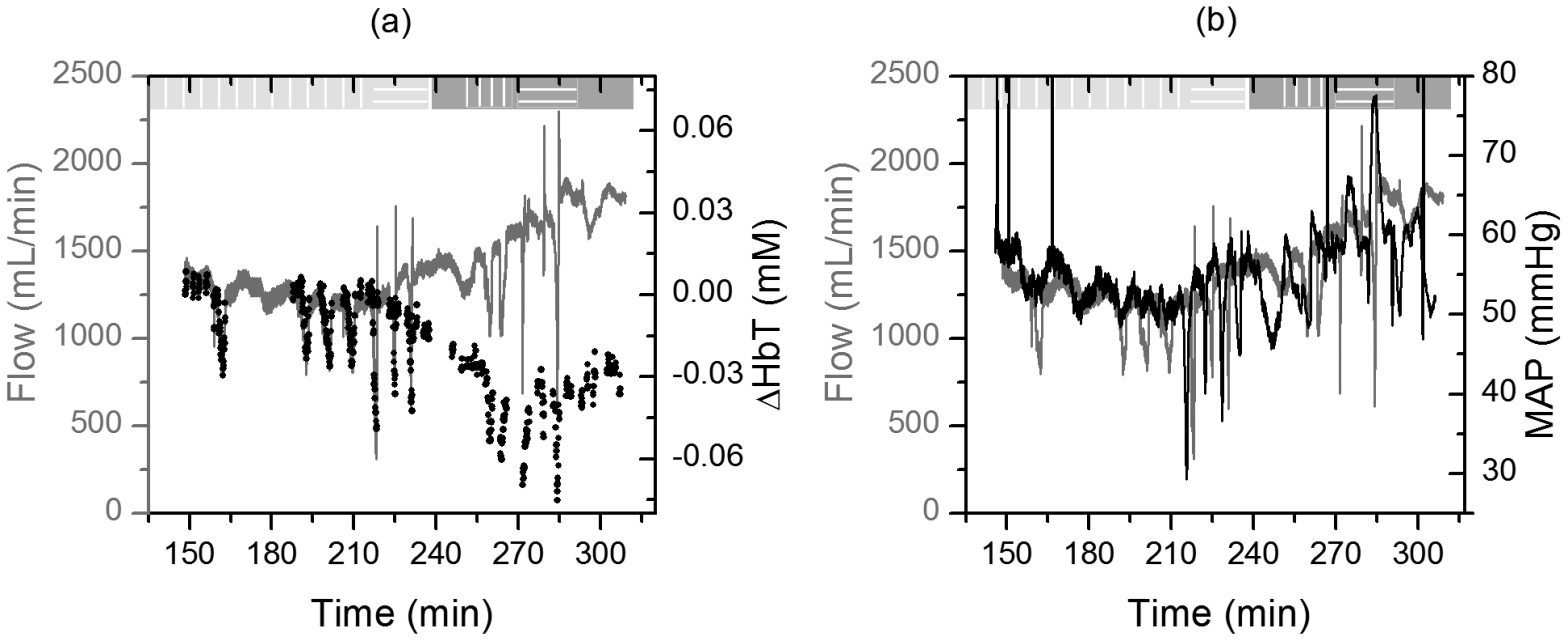
Hepatic flow changes. (Left) Changes in total hemoglobin concentration (ΔHbT, black dots) in hepatic tissue versus total hepatic flow measured by the addition of the HA and PV Transit-Time flowmeters' measurements (grey line). (Right) Total hepatic flow (grey) and mean arterial pressure (black) trends show that the increase in flow after t = 200 min is accompanied by an increase in the arterial pressure suggesting that a systemic response is responsible for that increase.

Figure 11-a shows the changes in hemoglobin concentration measured by the optical telemetry sensor and the total hepatic flow measured by the transit-time ultrasonic flowmeters. The two quantities correlate with high accuracy in the first 70 minutes of the study (t = 150–220 min) during which four HA occlusions were performed. After the first PV occlusion (t = 220 min), the signal from the optical telemetry sensor showed a slow decreasing trend in perfusion while the transit-time flowmeter showed an increase. This is due to the fact that the telemetry sensor is measuring tissue perfusion directly by looking at the tissue hemoglobin content while the ultrasonic transit-time flowmeter is measuring vascular flow changes. This discrepancy between the two can be due to a systemic response causing vasoconstriction that results in a decrease in microvasculature perfusion while increasing the blood flow in the central vasculature. Such a response can be triggered by a decrease in blood pressure. To verify this theory, we looked at the change in the Mean Arterial Pressure (MAP) shown in Figure 11-b. During the period of increase in the Transit-Time flowmeter signal, the MAP increased from 52 mmHg to more than 70 mmHg. We believe this event was triggered by the first portal venous occlusion that caused a decrease in venous return to the heart thereby causing a decrease in blood pressure to around 30 mmHg. This decrease in pressure is not seen during the first four occlusion events (HA occlusions) since the HA flow (350 mL/min in humans) is much lower than the PV flow (1100 mL/min, ∼22% of total cardiac output) [28]. In general, vasoconstriction is associated with an increase in MAP which supports our proposed explanation [28]. In addition, we looked at the telemetry sensor data from the hepatic artery probe, and they showed a similar increasing trend as measured by the Transit-Time flowmeter (Figure S7). This is an advantage of the employed technique since perfusion measurements are desired. Flow readings are usually used as an estimate of perfusion trends; however, in addition to perfusion, these measurements are affected by changes in blood pressure. Spectroscopy based techniques measure the real hemoglobin content in tissue which is essential to know the availability of nutrients and oxygen to cells.

**Figure 12:**
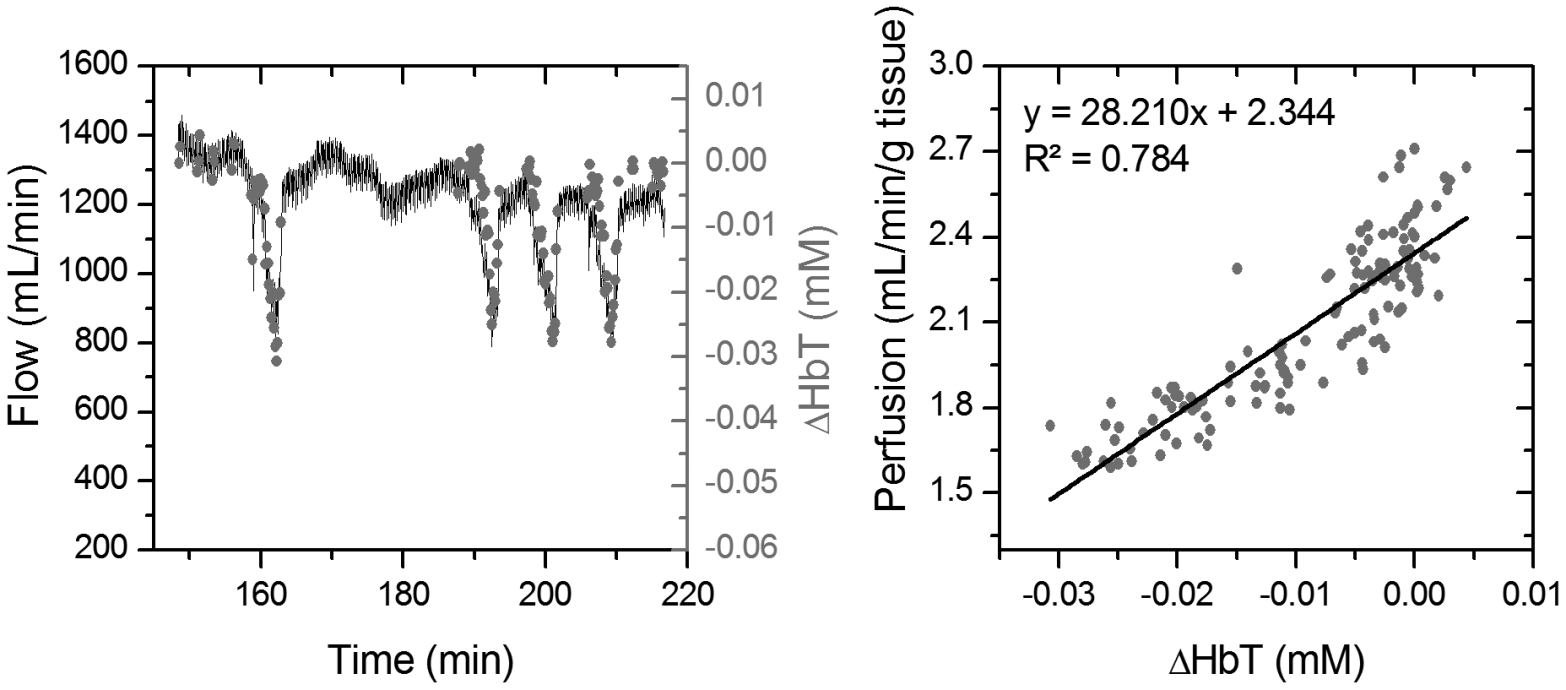
Hepatic flow changes prior to the increase in blood pressure. (Left) Total hemoglobin concentration (ΔHbT) and flow changes. (Right) Scatter plot of measured hemoglobin concentration change (ΔHbT) vs. tissue perfusion (flow normalized by liver weight).

To assess the accuracy of the perfusion measurements, we compared the readings from the telemetry sensor and the Transit-Time flowmeter prior to the increase in blood pressure (first 4 occlusions). The telemetry sensor was able to resolve perfusion changes with an RMSE of 0.135 mL/min/g of tissue (70.87 mL/min) as shown in Figure 12. Note that the standard deviation of the Transit-Time flowmeter measurements (0.09 mL/min/g of tissue ⇔ 47.6 mL/min) during the first baseline collection (t = 148–155 min) is on the same order as the RMSE of the telemetry sensor.

## Discussion

The oxygenation measurements obtained by the telemetry sensor agreed for the most part with the reference oxygen saturation measurements acquired by the vascular catheters. Specifically, the pulse oximetery signal was shown to track the defined quantity MOS which is a function of the flow and oxygenation of the HA and PV. The hepatic tissue supply oxygenation was measured with an RMSE of 2.82% for the range 72–100%. We were able to resolve venous oxygenation changes with an RMSE of 1.39%. This resolution went up to 3.93% when vascular occlusions were performed simultaneously with the hypoxia. Finally, we were able to measure perfusion changes with a resolution of 0.135 mL/min/g of tissue in the range of 1.5 to 3.0 mL/min/g. This resolution was similar to the variation of the baseline measurements by the reference flowmeters.

The main discrepancy between the oxygenation measurements obtained by the telemetry sensor and the reference oxygen saturation measurements acquired by the vascular catheters occurred during HA occlusions where the telemetry sensor showed a decrease in the venous oxygen saturation while the central venous catheter did not show any change. We believe that the short duration of occlusions on the HA caused a local drop in the intrahepatic venous oxygenation which was not reflected in the systemic data. Note that some reports have stated concerns about using NIRS to estimate oxygenation changes during changes in tissue hemoglobin content [29] while others use this method to assess desaturation during occlusions [30]. This makes it more important to always report the total hemoglobin concentration (HbT) along with the oxygenation level so that the user is aware of the possibility of low accuracy in oxygenation measurements during low perfusion periods.

During vascular occlusions, the measured hemoglobin concentration changes correlated with the hepatic flow (R^2^∼0.78). During these occlusions, HbT levels decreased, while Hb concentration increased and HbO_2_ concentration decreased. This agrees with previously reported findings by El-Desoky *et al.* that measured hemoglobin changes in porcine hepatic tissue using a commercial bench-top NIRS system [21]. The RMSE in tracking tissue perfusion was found to be 0.13 mL/min/g. We believe that part of this error is caused by the comparison of direct perfusion measurements from the telemetry system to flow levels measured by the reference vascular flowmeters. Soft tissue acts as a capacitor and quick changes seen in the vascular flow can sometimes be delayed and filtered by the tissue. Note that the described sensor measures changes in tissue hemoglobin content and not perfusion. These two quantities may differ in some cases such as during venous congestion. However, coupling this measurement with the oxygenation data gives the medical care provider the information needed to detect these cases. For example, the increase in tissue hemoglobin content during venous congestion is accompanied by a drop in tissue oxygen saturation which indicates pooling of venous blood within tissue.

The results presented in this manuscript are a first realization of a wireless optical sensor for monitoring liver tissue hemodynamics. We believe this technology can lead to a fully implantable device to monitor patients in the 14 days period following transplant. Over 55% of rejections take place in these two weeks [3]. Following that period, the device can be explanted in a minimally invasive surgery. The device can be implanted either intraperitoneally and fixed on liver tissue, or submdermally with a probe linking the main unit to hepatic tissue. Many challenges need to be addressed before this prototype can be translated into a viable clinical product. One of these challenges is the power source. As mentioned earlier, the current prototype runs on a lithium-ion battery. The current battery is capable of running a single sensor for almost 14 days (13.89 days) with one reading (30 seconds of data) every 5 minutes. Lowering the power consumption of the device and/or finding alternative ways to power it, such as inductive coupling, can reduce the size of the battery and lead to a smaller implant. Another challenge is the coating of the device. The encapsulation of the implanted sensor due to innate host response can lead to difficulties in the sensor removal which may lead to injury. Similar to other implanted electronic devices (i.e. pacemaker), the sensor has to be placed in a sealed enclosure such as a titanium case to protect the electronics. The casing can then be coated with a “biocompatible” polymer if needed such as polyethylene glycol. Finally, motion artifacts and being able to account for those is another challenge. Our current design implements a set of band-pass filters that allow us to filter out the majority of low frequency (<0.6 Hz) artifacts, such as respiratory motion and other slow processes, and high frequency (>15 Hz) noise. In addition, we have developed multiple processing techniques to use the additional wavelengths to account for artifacts due to motion and other confounding variables [31], [32].

## Conclusions

In this paper, NIRS combined with photoplethysmography (PPG) was used to demonstrate, for the first time, simultaneous, *in vivo* wireless, measurements of hepatic perfusion, and oxygenation levels. The combination of these two techniques does not require any additional hardware to the traditional PPG sensors and offers the advantage of measuring simultaneously oxygen supply (MOS) and consumption (SvO_2_). The ability to measure these parameters simultaneously ultimately gives physicians a more complete picture of the graft hemodynamics providing some confidence that this approach has potential for assessing the organ function in real time and detecting complications at an early stage.

## Supporting Information

Figure S1 **CAD drawings of the probes.** a-Suture holders for the parenchymal, HA, and PV probes (left to right). b & c- CAD drawing of a parenchymal and a vascular probe respectively.
(TIF)

Figure S2 **The optical sensors used in the studies.** (Left) CAD drawing of the electronics box showing the PCBs inside. (Right) Picture of the telemetry system showing the sensor, probes, and data acquisition software.
(TIF)

Figure S3 **Laser Doppler and transit time Doppler perfusion and flow measurements.** Scatter plots of the Laser Doppler data versus HA flow (left), PV flow (middle), and total hepatic flow (right). The data shows that the LD data correlates best with the HA flow suggesting that the system was probing a branch of the HA.
(TIF)

Figure S4 **Reference flow measurements.** Data from the transit time flowmeters (left axis) and Laser Doppler flowmeter (right axis). The time segments highlighted in red correspond to HA occlusion studies while the blue segments correspond to PV occlusion studies.
(TIF)

Figure S5 **Oximetry catheters' data.** Arterial and venous oxygen saturation from experiment 1 (left) and 2 (right).
(TIF)

Figure S6 **Comparison of the measured hemoglobin oxygenation index (ΔHbD) to the arterial and venous oxygen saturation levels.** (Left) Correlation between ΔHbD and the measured oxygenation levels. (Right) Calibrated ΔHbD can predict SvO2 with a higher degree of confidence (R2 = 0.99) when using a multiple linear regression taking account for both supply and venous oxygenation.
(TIF)

Figure S7 **Total hemoglobin concentration (ΔHbT) as measured by the hepatic artery probe.**
(TIF)

Supporting Information S1 (DOCX)
