# Supplementary figures and images for: Wireless Monitoring of Liver Hemodynamics In Vivo
Source: PLoS One. 2014 Jul 14;9(7):e102396. doi: 10.1371/journal.pone.0102396 (PMC4097065; doi:10.1371/journal.pone.0102396)

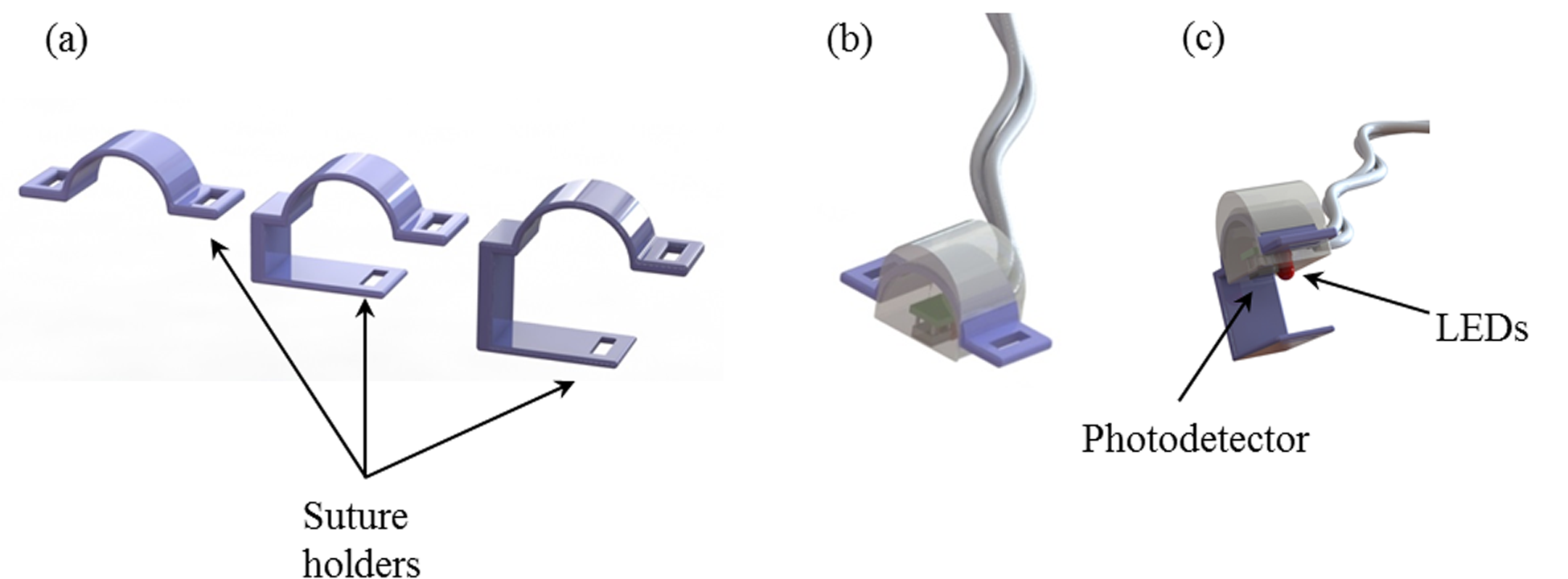

Supplement: Figure S1 — CAD drawings of the probes. a-Suture holders for the parenchymal, HA, and PV probes (left to right). b & c- CAD drawing of a parenchymal and a vascular probe respectively. (TIF) [file pone.0102396.s001.tif]

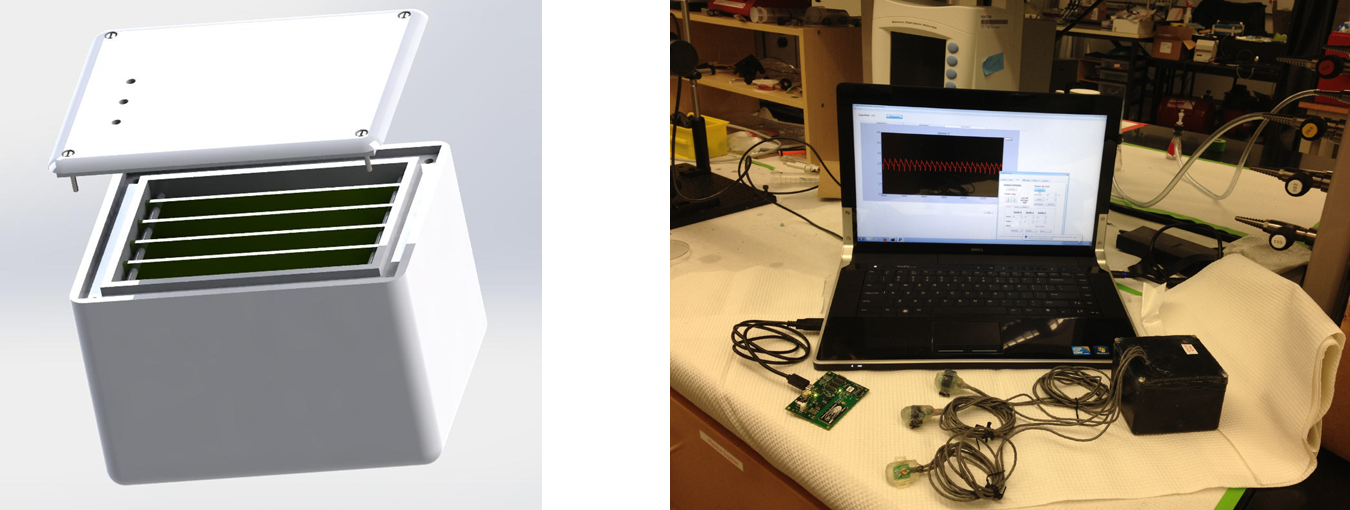

Supplement: Figure S2 — The optical sensors used in the studies. (Left) CAD drawing of the electronics box showing the PCBs inside. (Right) Picture of the telemetry system showing the sensor, probes, and data acquisition software. (TIF) [file pone.0102396.s002.tif]

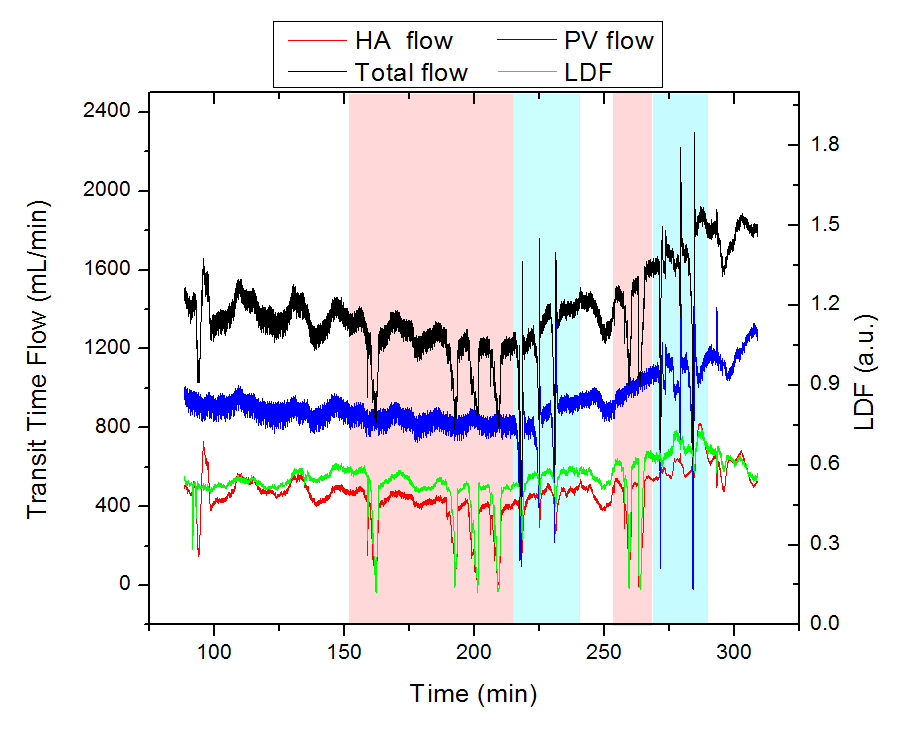

Supplement: Figure S4 — Reference flow measurements. Data from the transit time flowmeters (left axis) and Laser Doppler flowmeter (right axis). The time segments highlighted in red correspond to HA occlusion studies while the blue segments correspond to PV occlusion studies. (TIF) [file pone.0102396.s004.tif]

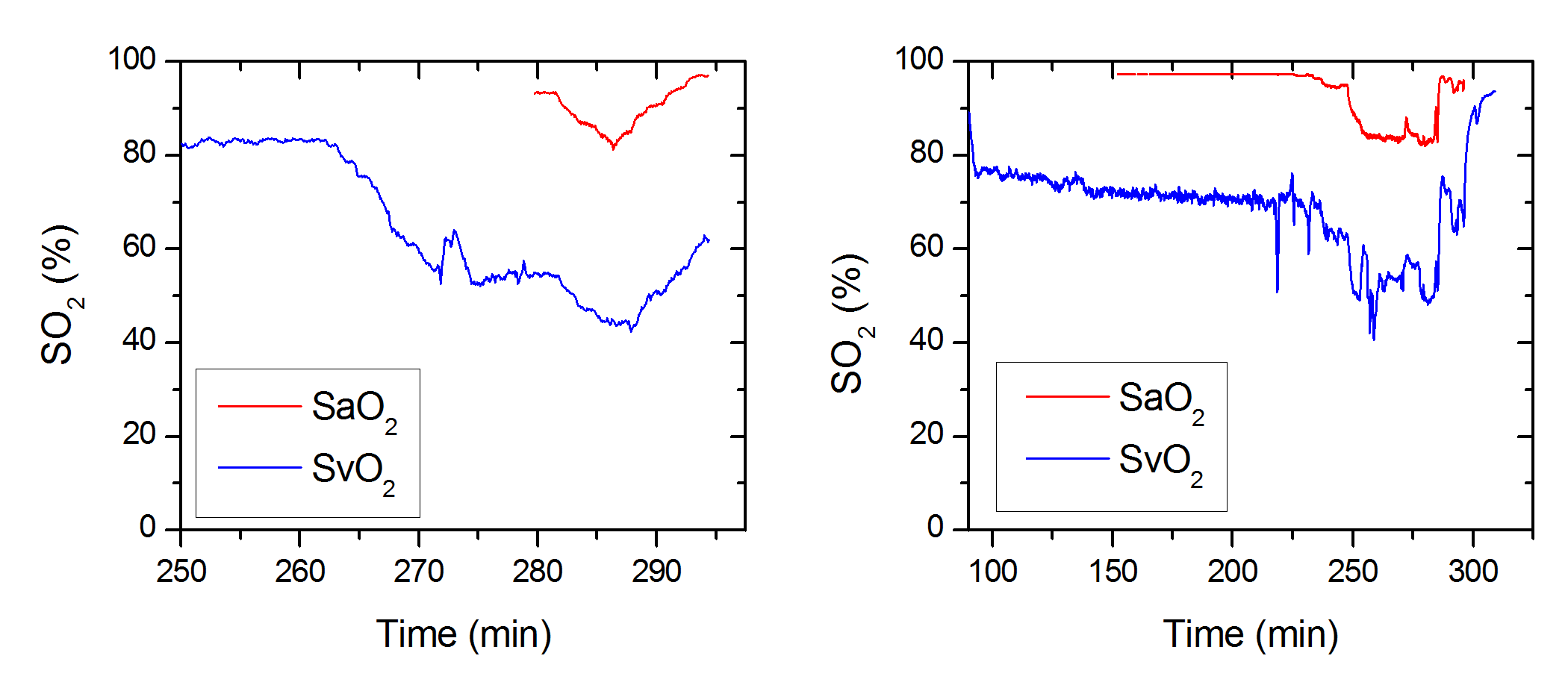

Supplement: Figure S5 — Oximetry catheters' data. Arterial and venous oxygen saturation from experiment 1 (left) and 2 (right). (TIF) [file pone.0102396.s005.tif]

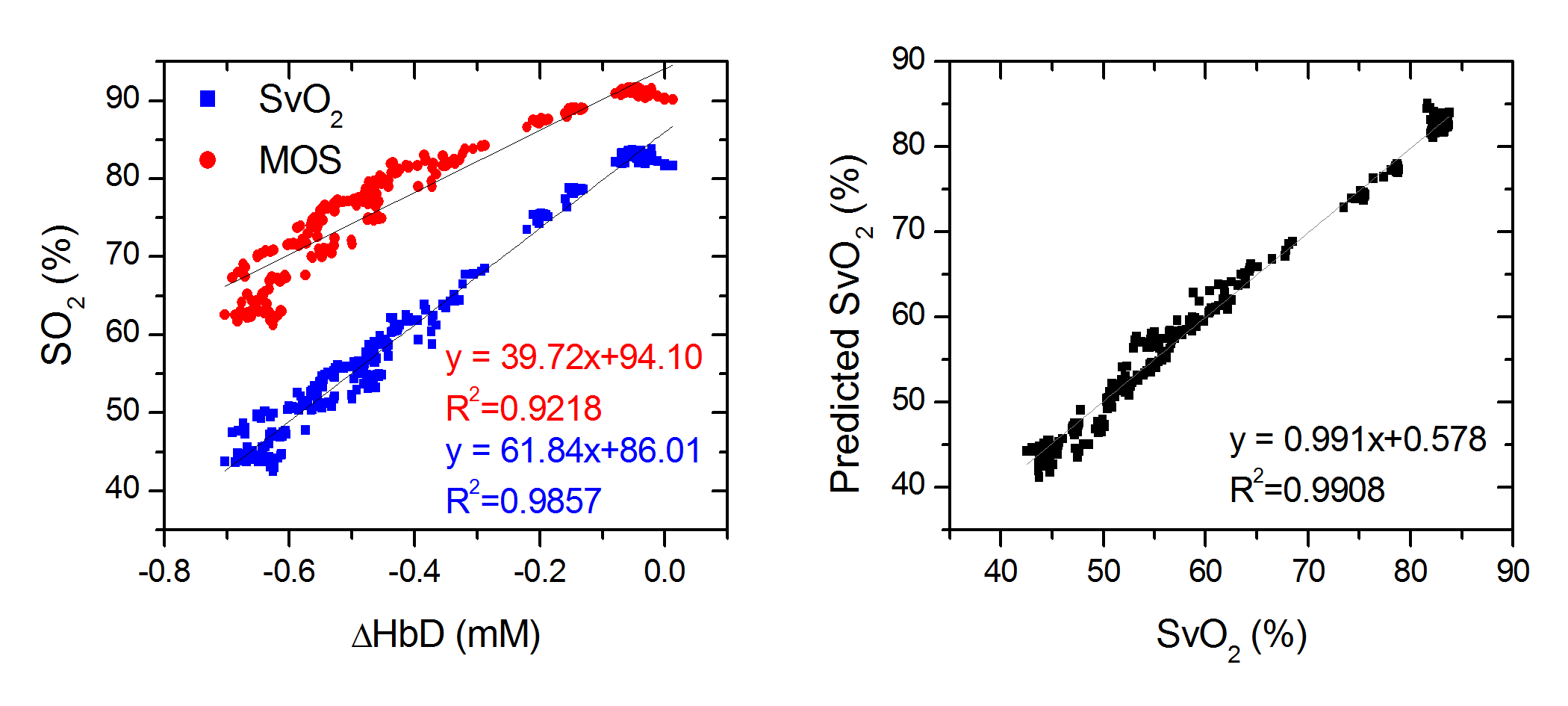

Supplement: Figure S6 — Comparison of the measured hemoglobin oxygenation index (ΔHbD) to the arterial and venous oxygen saturation levels. (Left) Correlation between ΔHbD and the measured oxygenation levels. (Right) Calibrated ΔHbD can predict SvO2 with a higher degree of confidence (R2 = 0.99) when using a multiple linear regression taking account for both supply and venous oxygenation. (TIF) [file pone.0102396.s006.tif]
