# Supplementary material for: Wireless Monitoring of Liver Hemodynamics In Vivo
Source: PLoS One. 2014 Jul 14;9(7):e102396. doi: 10.1371/journal.pone.0102396 (PMC4097065; doi:10.1371/journal.pone.0102396)
Supplement: Supporting Information S1 — (DOCX) [file pone.0102396.s008.docx]

**Supplementary information**

This document contains extra details about the paper “Wireless Monitoring of Liver Hemodynamics *In Vivo*” that were not included in the manuscript.

1. Probe fabrication & sensor encapsulation:

All sensor probes were coated with Polydimethylsiloxane (PDMS) to avoid water leakage into the electronics. To do that, the Printed Circuit Board (PCB) with the Light Emitting Diodes (LEDs) and photodetector was secured in a 3D printed mold and PDMS was poured on it. The mold was then placed in an oven for roughly 1 hour at 65°C to cure the PDMS. The PDMS coated electronics were placed in a different mold containing one of three 3D printed suture-holders designed to secure the probes in place (Figure S1). PDMS was poured into this mold and placed again in the oven for an extra hour at 65°C. The probes were left overnight at room temperature before any testing was performed. All PDMS was mixed with a curing agent at a volumetric ration of 10:1 and degassed for 60 minutes to avoid air pockets. The PDMS was degassed again after being poured in the mold to make sure no air bubbles were introduced in the process. The probes were coated with an additional thin layer of electronic grade silicone to avoid any leakage. Prior to the animal studies, all probes were placed in a water bath overnight to test for leakage.

Figure S1-a shows CAD models of the three different suture holders used in the parenchymal (left) and vascular probes (middle and right). The vascular probes were made in two different sizes to be secured around the hepatic artery and portal vein that differ in diameter (HA ~ 5 mm and PV ~ 8-10 mm).

All sensor electronics were enclosed in a 3D printed box (figure S2). The box was coated with a thin layer of electronic grade silicone.

1. Reference Measurements:

The data from the Laser Doppler (LD) Perfusion monitor did not correlate well with tissue perfusion changes. In both studies we had two LD monitors placed on the hepatic tissue: a surface probe and a needle probe. The probes were adjusted and moved to various spots on the tissue to be able to pick up a Doppler signal. We were not able to get a signal with the needle probe. The surface probe gave a good Doppler signal. However, further analysis showed that the signal by that probe correlated well with the hepatic artery flow but not with the portal venous flow. We believe that the LD was probing a branch of the hepatic artery embedded in tissue. The data from the LD was not used as a reference, and instead we used the total hepatic flow obtained by the two Transit-Time Ultrasound flowmeters. Figure S3 shows the correlation between the LD output and the HA and PV flow measured by the Transit-Time flowmeters. As mentioned, the LD signal correlates with the HA flow with a coefficient of determination (R^2^) of 0.8 while the correlation with PV flow showed an R^2^ of 0.1. The time pattern of the measured signals is shown in Figure S4. Note that during the PV occlusion studies (Figure S4, blue segments), the HA occluders were open (not inflated). All changes in HA flow during these segments are due to a systemic response.

Figure S5 shows the raw data from the arterial and venous oxygenation catheters from both studies described in the manuscript. The catheters used do not measure oxygenation levels above 97.5% and that is why some data for the arterial catheter is missing from the graph below.

1. Data processing:

Figure S6 shows the correlation between the measured hemoglobin oxygenation index and the different reference oxygenation measurements (MOS and SvO_2_). The data show that ΔHbD correlates best with a combination of the two which is expected since the collected light probe both the venous and arterial blood.

The total blood volume on the hepatic probe is shown in Figure S7 below. Note that this probe was palced on hepatic tissue surrounding the hepatic artery at the entrance to the liver. The blood supply to this tissue should be dominated by the hepatic artery. Unlike the probes on the peripheral tissue, this probe showed an increase in the average tissue blood content after the portal vein occlusions which supports our conclusion of vasoconstriction triggered by the drop in blood pressure.
